# Improving diagnosis of mitochondrial fatty-acid oxidation disorders

**DOI:** 10.1038/s41431-022-01260-1

**Published:** 2023-01-05

**Authors:** Christine Vianey-Saban, Alain Fouilhoux, Jerry Vockley, Cécile Acquaviva-Bourdain, Nathalie Guffon

**Affiliations:** 1 Biochemical and Molecular Biology Laboratory, Metabolic Inborn Errors of Metabolism Unit, Groupement Hospitalier Est, CHU de Lyon, 69500 Bron, France; 2 National Reference Centre for Hereditary Metabolic Diseases, Groupement Hospitalier Est, CHU de Lyon, 69500 Bron, France; 3 UPMC Children’s Hospital of Pittsburgh, Genetic and Genomic Medicine, Center for Rare Disease Therapy, Department of Pediatrics, University of Pittsburgh School of Medicine, Pittsburgh, PA 15224 USA

**Keywords:** Diagnostic markers, Metabolic disorders, Energy metabolism, Fat metabolism

## Introduction

**Fig. 1:**
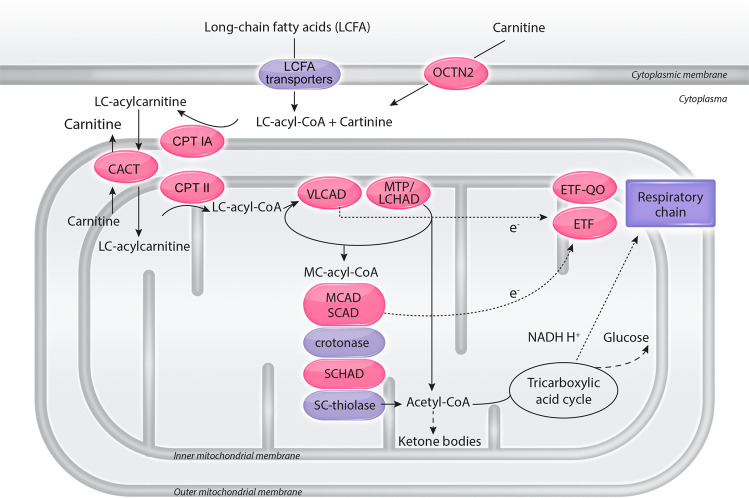
A simplified scheme of mitochondrial fatty acid oxidation. Most fatty acid oxidation occurs in the mitochondria. Medium- and short-chain fatty acids directly enter the mitochondria. Long-chain fatty acids (LCFA) circulate in plasma bound to albumin. They are carried across the plasma membrane via several LCFA transport systems, CD36 (cluster of differentiation 36) being probably the most important. LCFA must be activated to coenzyme A and transferred to carnitine (CPT IA) to cross the inner mitochondrial membrane (CACT). They are then transferred back to CoA esters in the mitochondrial matrix (CPT II). Beta-oxidation is catalysed by enzymes with different fatty acid chain length specificity (VLCAD, MTP, MCAD, SCAD, crotonase, SCHAD, and SC-thiolase). Electrons (e^−^) are passed to the respiratory chain either directly or via transfer proteins (ETF, ETF-QO). Acetyl-CoA can be oxidised in the tricarboxylic acid (Krebs) cycle or, in the liver, used to synthetize ketone bodies. Fatty acid oxidation disorders mentioned in this manuscript are labelled in red. Crotonase (also named short-chain enoyl-CoA hydratase) deficiency has a different clinical presentation, like mitochondrial cytopathies. No deficiency of LCFA transporter(s) and SC-thiolase has been identified so far. CACT carnitine acylcarnitine translocase, CPT IA carnitine palmitoyl transferase IA, CPT II carnitine palmitoyl transferase II, ETF electron transfer flavoprotein, ETF-QO electron transfer flavoprotein ubiquinone oxidoreductase, LC long-chain, LCFA long-chain fatty acids, LCHAD long-chain 3-hydroxyacyl-CoA dehydrogenase, MC-acyl-CoA medium-chain acyl-CoA, MCAD medium-chain acyl-CoA dehydrogenase, MTP mitochondrial trifunctional protein, OCTN2 high affinity sodium-dependent carnitine transporter, SCAD short-chain acyl-CoA dehydrogenase, SCHAD short-chain 3-hydroxyacyl-CoA dehydrogenase, SC-thiolase short-chain acyl-CoA thiolase, VLCAD very long-chain acyl-CoA dehydrogenase.

Mitochondrial Fatty Acid Oxidation Disorders (FAOD, Table 1, Fig. 1) include 12 genetically distinct metabolic disorders, inherited as an autosomal recessive trait, with an estimated cumulative incidence from 1:6,500 to 1:110,000 [1]. Their clinical presentation ranges from fatal acute hypoglycaemic crises in neonates to less severe later onset conditions characterised by myalgia and exercise intolerance. Symptoms differ for each, and phenotypic diversity extends even to patients bearing identical genetic variants [2]. In the most severe cases, neonatal presentation includes recurrent episodes of hypoketotic hypoglycaemic encephalopathy, liver dysfunction, often cardiac dysfunction, and sometimes congenital malformations. Therefore, it is important to rapidly implement emergency protocols for the acute management of metabolic crises [3].

**Table 1:** Main clinical symptoms and routine blood test results when symptomatic of mitochondrial fatty acid oxidation disorders (FAOD).

Disorder	Full-name	Gene(s)	Age of onset of first symptoms			Clinical symptoms							Routine blood test results						
			Neonates- Infants <2 years	2–11 years	>11 years	Sudden death *or* Life threatening events	Liver. *hepatomegaly, steatosis*	Muscle. *weakness, pain, rhabdomyolysis*	Heart. *hypertrophic cardiomyopathy without arterial hypertension, heart failure, arrythmia*	Ocular. *retinitis pigmentosa*	Neurological. *peripheral neuropathy, seizures*	Renal. *tubular acidosis*	Hypoketotic hypoglycaemia	↑ Ammonia (*neonates / infants*)	↑ ALAT +/− ASAT	↑ Lactic acid (*neonates / infants*)	↑ Creatine kinase	Anaemia	Hyper-insulinism
CACT	Carnitine-acylcarnitine translocase deficiency	*SLC25A20*	+			+	+	+	+			+	+	+	+	+	+		
CPT IA	Carnitine palmitoyl transferase type IA deficiency	*CPT1A*	+				+					+	+	+	+				
LCHAD^a^	Long-chain 3-hydroxyacyl-CoA dehydrogenase deficiency	*HADHA*	+			+	+	+	+	+	+ (neuropathy)		+		+	+	+		
MAD	Multiple acyl-CoA dehydrogenase deficiency (ETF or ETF-QO deficiency)	*ETFA, ETFB or ETFDH*	+	+		+	+	+	+				+	+	+	+	+		
SCHAD	Short-chain 3-hydroxyacyl-CoA dehydrogenase deficiency	*HADH*	+	+									+						+
CTD^b^	Carnitine OCTN2 transporter deficiency	*SLC22A5*	+	+	+	+	+	+	+ (heart failure)				+	+	+		+	+	
CPT II	Carnitine palmitoyl transferase type II deficiency	*CPT2*	+	+	+	+	+	+	+				+	+	+	+	+		
VLCAD	Very long-chain acyl-CoA dehydrogenase deficiency	*ACADVL*	+	+	+	+	+	+	+				+		+	+	+		
MTP^a^	Mitochondrial trifunctional protein deficiency	*HADHA* *HADHB*	+	+	+	+	+	+	+	+	+ (neuropathy)		+		+	+	+		
MCAD^b^	Medium-chain acyl-CoA dehydrogenase deficiency	*ACADM*	+	+	+	+	+		+/− (in adults)				+	+	+	+	+		
SCAD^b^	Short-chain acyl-CoA dehydrogenase deficiency	*ACADS*	+	+	+			+ (weakness)			+ (seizures)		+						
RR-MAD	Riboflavin responsive multiple acyl-CoA dehydrogenase deficiency	*ETFDH*		+	+			+									+		

*↑*Indicating abnormally elevated results from these blood tests, *ALAT* alanine aminotransferase, *ASAT* aspartate aminotransferase.

^a^During pregnancy, mothers of foetuses affected with LCHAD or MTP can suffer from Haemolysis, Elevated Liver enzymes and Low Platelets (HELLP) syndrome or Acute Fatty Liver of Pregnancy (AFLP).

^b^Asymptomatic patients have been reported.

Later onset FAOD are often associated with chronic symptoms such as hypotonia, exercise intolerance, or hepatic dysfunctions. Retinopathy and peripheral neuropathy are specific to long-chain 3-hydroxyacyl-CoA dehydrogenase (LCHAD) and mitochondrial trifunctional protein (MTP) deficiency [2, 4, 5]. Fasting or other physiologic stresses can lead to crises of muscle breakdown (rhabdomyolysis), and cardiac muscle damage [2]. These symptoms greatly impact quality of life. Moreover, left undiagnosed, patients of all ages are exposed to the risk of fatal metabolic decompensations [2]. Access to simple, effective management strategies and dietary therapies can greatly improve quality of life [3, 6].

New-born screening (NBS) programs improve early diagnosis of FAOD [1], but ethical and economic factors still play an important role in its implementation, limiting access in some locations [7]. As such, differential diagnosis of FAOD remains important, to initially recognise symptoms and later to account for possible normal biochemical testing that may occur when blood samples are collected from patients not in catabolism [8].

The objective of this manuscript is to indicate appropriate specimens to be collected and analysed, and to propose diagnostic algorithms according to symptoms. These algorithms are divided into three categories by age and are designed as stand-alone tools to facilitate the prompt diagnosis of mitochondrial FAOD by primary caregivers and thus accelerate the accurate referral of patients to specialists. Where necessary, differential diagnoses are indicated so that physicians may seek further resources.

## Laboratory testing

Table 2 outlines the main laboratory tests involved in FAOD diagnosis. Initially, routine blood analyses that can direct further testing should be conducted, e.g., anaemia in carnitine OCTN2 transporter deficiency (CTD). Routine testing, however, may appear normal unless samples were taken during an acute metabolic decompensation. At such times, hypoketotic hypoglycaemia may be observed, with increased levels of lactic acid, ammonia, and/or creatine kinase.

**Table 2:** Laboratory investigation of mitochondrial fatty acid oxidation disorders (FAOD).

Laboratory tests	Specimen type	Special conditions for sampling	Expected abnormalities in FAOD
**Routine tests**			
Glucose	Plasma or serum	Acute episode	<3 mmol/L
Ketone bodies	Urine: dipstick Blood: enzymatic measurement or blood metre	Acute episode Blood immediately deproteinised if enzymatic measurement	When glycaemia <3 mmol/L - Urine < ++ - Blood <1 mmol/L
Ammonia	Plasma	Acute episode Blood brought on ice within 10 min to the laboratory for testing	>100 μmol/L (mainly new-borns and infants)
Transaminases (ASAT, ALAT)	Plasma or serum	Acute episode	ASAT > ALAT ASAT, ALAT > 200 UI/L
Lactic acid	Blood	Acute episode	>2 mmol/L
Creatine kinase (CK)	Plasma or serum	Acute episode	>1000 UI/L
Complete cell count	Blood (EDTA)		Anaemia (CTD)
Insulin C-peptide	Plasma	Acute episode	When glycaemia <3 mmol/L - Insulin ≥2 mUI/L **(SCHAD)** - C-peptide ≥165 pmol/L)
**When the patient is in non-catabolic clinical conditions, results for routine tests in the range of controls do not exclude a FAOD**			
**Biochemical genetic tests**			
Acylcarnitine profile	Plasma or dried blood spot (DBS)	Acute episode or fasting state: in adults >14 hours (mandatory), in children depends on age	- CTD: ↓ C_0_ and all acylcarnitines - CPT IA: ↑ **C**_**0**_ ↓ C_16_ C_18:1_ *(dried blood spot)* - CPT II, CACT (early onset): ↑ C_12_ C_14_ **C**_**16**_ **C**_**18:1**_ C_16-DC_ C_18:1-DC_ - CPT II (late onset): ↑ **C**_**16**_ **C**_**18:1**_ *(plasma)* - LCHAD / MTP: ↑ **C**_**14-OH**_ C_16_ **C**_**16-OH**_ C_18:1_ **C**_**18:1-OH**_** C**_**18-OH**_ - VLCAD: ↑ **C**_**14:1**_ C_16_ C_18:1_ - MCAD: ↑ **C**_**6**_** C**_**8**_** C**_**10:1**_ C_10_ (C8/C10>2) - MAD, riboflavin disorders (mainly FAD synthase, MFT): ↑ **C**_**4**_** C**_**5**_ C_6_ C_8_ **C**_**10**_ C_5-DC_ C_12_ C_14_ C_16_ C_18:1_ (possibly not all acylcarnitines) - SCAD: ↑ **C**_**4**_ - SCHAD: ↑ **C**_**4-OH**_
Free and total carnitine	Plasma Urine if plasma free carnitine <5 μmol/L		- CTD: plasma free and total carnitine <5 μmol/L, urine free and total carnitine >5 mmol/mol of creatinine - Other FAOD except CPT IA: in general, plasma free carnitine <20 μmol/L, total-free carnitine >15 μmol/L - CPT IA: plasma free carnitine >50 μmol/L in DBS (can be normal in plasma) **Plasma carnitine levels in the range of controls do not exclude a FAOD**
Organic acid profile	Urine	Acute episode or fasting state	- All FAOD: saturated and unsaturated dicarboxylic acids +/− 3-hydroxydicarboxylic acids - MCAD: same all FAOD + hexanoylglycine, suberylglycine, phenylpropionylglycine - MAD, riboflavin disorders (mainly FAD synthase, MFT): same MCAD + isobutyrylglycine, 2-methylbutyrylglycine, isovalerylglycine, ethylmalonic acid, 2-hydroxyglutaric acid +/− glutaric acid - SCAD: ethylmalonic acid, methylsuccinic acid +/− butyrylglycine - SCHAD: 3-hydroxyglutaric acid **A normal urinary organic acid profile does not exclude a FAOD**
In vitro flux studies	Blood Intact cultured skin fibroblasts	Blood sample must reach the laboratory in less than 48 hours Contact the laboratory for skin biopsy conditions	Abnormal de novo synthesis of ^2^H-acylcarnitines from ^2^H-palmitate and L-carnitine or decreased oxidation rate of ^3^H or ^14^C labelled fatty acids
Specific enzyme activity measurement		Limited number of laboratories in the world	Performed if the pathogenicity of the identified variant(s) has not been demonstrated
**Molecular genetic tests**			
Single gene (Sanger method)	Blood (EDTA). Eventually frozen tissues (muscle, liver, …) or cultured skin fibroblasts		Detection of pathogenic variants (homozygous or compound heterozygous)
Gene panel (Next Generation Sequencing [NGS])			
Whole exome or genome sequencing (ultra-high throughput sequencing)			

*C*_*0*_ free carnitine, *CACT* carnitine acylcarnitine translocase deficiency, *CPT IA* carnitine palmitoyl transferase IA deficiency, *CPT II* carnitine palmitoyl transferase II deficiency, *CTD* carnitine OCTN2 transporter deficiency, *FAD* flavin adenine dinucleotide, *FAOD* fatty acid oxidation disorders, *LCHAD* long-chain 3-hydroxyacyl-CoA dehydrogenase deficiency, *MAD* multiple acyl-CoA dehydrogenase deficiency (ETF or ETF-QO deficiency), *MCAD* medium-chain acyl-CoA dehydrogenase deficiency, *MFT* mitochondrial FAD transporter, *MTP* mitochondrial trifunctional protein deficiency, *SCAD* short-chain acyl-CoA dehydrogenase deficiency, *SCHAD* short-chain 3-hydroxyacyl-CoA dehydrogenase deficiency, ↑ indicating abnormally elevated results from these blood tests, ↓ indicating abnormally decreased results from these blood tests. The acylcarnitines in bold characters are the most clinically relevant species for each disorder.

Next, biochemical analysis of acylcarnitine profile by tandem mass spectrometry in blood specimens is central for the diagnosis of all FAOD and serves as the basis for NBS. Results from these assays allow to identify the predominant fatty acid derived metabolites and to characterise carnitine levels. It is again crucial to note that blood samples must be collected during an acute crisis or after fasting, as acylcarnitine profiles can normalise in anabolic state, leading to negative results [8].

High levels of free carnitine, especially in dried blood spots (DBS), should trigger consideration of carnitine palmitoyl transferase type IA deficiency (CPT IA). Very low levels of plasma carnitine (<5 µmol/L), contrasting with a urine carnitine level >5 mmol/mol of creatinine suggests CTD. For patients with low carnitine levels and non-specific acylcarnitine profiles, two options exist. L-carnitine supplementation can be given with subsequent testing of a new fasting blood sample, or blood cells (or eventually cultured skin fibroblasts) may be used for subsequent in vitro flux studies to measure the rate of fatty acid oxidation [9].

If the blood specimens have been collected post-mortem, acylcarnitine profiles can be non-informative, and could induce an incorrect diagnosis [8]. Thus, leftover samples collected during the acute decompensation, before death, should be sought to achieve highest confidence in the results of biochemical genetic tests.

Acylcarnitine profiles can be diagnostic (Table 2) [8, 10] and indicate which gene(s) to analyse to further confirm the diagnosis. If next-generation sequencing (NGS) analyses are available, physicians may choose to advance rapidly to this step, and test several candidate genes simultaneously. Elsewhere, targeted Sanger sequencing of both alleles of suspected gene(s) remains a robust technique. When variants of uncertain significance are identified, in vitro flux studies or measurement of enzyme activities are necessary to provide definitive diagnosis.

## Infants and young toddlers, <2 years old (Fig. 2)

Severe cases often present as acute and sometimes fatal crises of hypoketotic hypoglycaemia, associated with hepatic failure, hyperammonaemia (Reye-like syndrome) and/or cardiac symptoms (arrhythmia, cardiomyopathy), and possibly congenital malformations (mainly renal cysts and neuronal migration defects). Other cases present with milder, non-specific symptoms such as gross motor or language delay, and, over time, hypotonia and failure to thrive. Fasting and protein-induced hypoglycaemia with hyperinsulinism points to short-chain 3-hydroxyacyl-CoA dehydrogenase deficiency (SCHAD).

**Fig. 2:**
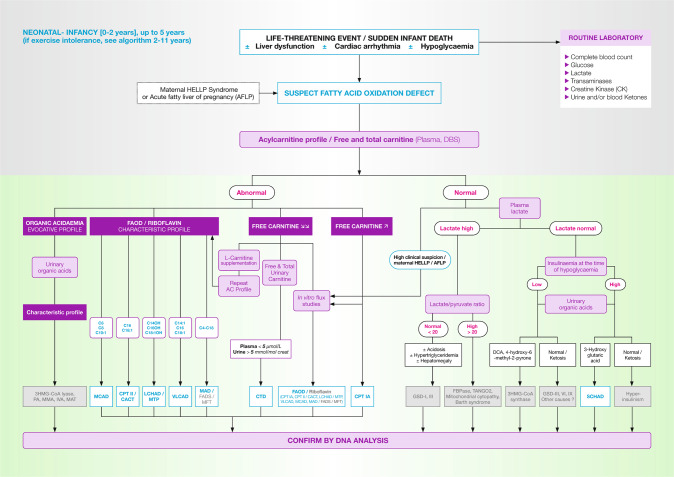
FAOD (fatty acid oxidation disorders) diagnosis in infants. 3HMG-CoA lyase 3-hydroxy-3-methylglutaryl-CoA lyase deficiency, 3HMG-CoA synthase 3-hydroxy-3-methylglutaryl-CoA synthase deficiency, AC acylcarnitine, AFLP acute fatty liver of pregnancy, CK creatinine kinase, DBS dried blood spot, DCA dicarboxylic acids, FAD flavine adenine dinucleotide, FADS FAD synthase deficiency, FBPase fructose 1,6 biphosphatase deficiency, GSD glycogen storage disorder, HELLP haemolysis, elevated liver enzymes, low platelet count syndrome, IVA isovaleric acidaemia, MAT mitochondrial acetoacetyl-CoA thiolase deficiency, MFT mitochondrial FAD transporter deficiency, MMA methylmalonic acidaemia, PA propionic acidaemia, *TANGO2*: transport and Golgi organisation 2 deficiency. The definitions for fatty acid oxidation disorders abbreviations (in blue) are given in Table 1.

Specimens should be collected during an acute crisis or after fasting for analysis of the blood acylcarnitine profile and urine organic acids to differentiate organic acidaemias. For patients with normal initial blood acylcarnitine profiles, medical history can provide additional insight. Maternal haemolysis, elevated liver enzymes and low platelets (HELLP) syndrome or acute fatty liver of pregnancy (AFLP) are seen especially in mothers carrying a foetus affected with MTP/LCHAD, and this history should trigger additional studies for these disorders including in vitro flux studies, or directly molecular genetic testing, depending on local facilities.

## Childhood, from age 2–11 years (Fig. 3)

Affected children <5 years of age can present severe hypoketotic hypoglycaemia and/or cardiac symptoms. Differential diagnosis in this setting is the same as for younger children.

**Fig. 3:**
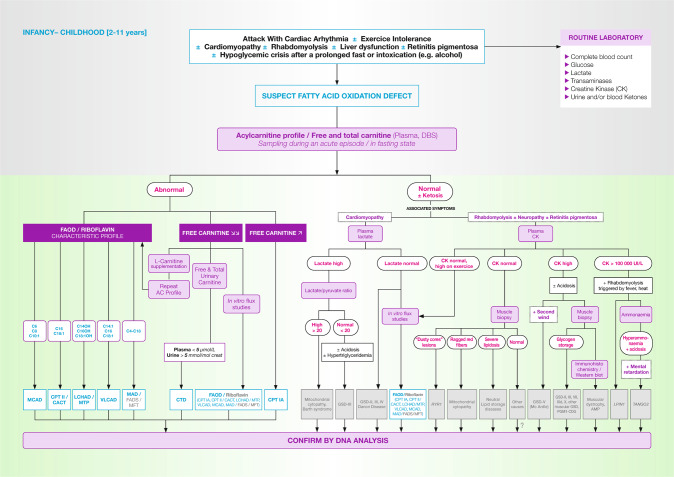
FAOD (fatty acid oxidation disorders) diagnosis in children. AC acylcarnitine, AMP adenosine monophosphate deaminase deficiency, CK creatinine kinase, DBS dried blood spot, FAD flavine adenine dinucleotide, FADS FAD synthase deficiency, GSD glycogen storage disorder, *LPIN1* phosphatidate phosphatase-1 deficiency, MFT mitochondrial FAD transporter deficiency, musc. GSD other muscular glycogen storage disorders, PGM1-CDG phosphoglucomutase 1 deficiency, *RYR1* ryanodine receptor 1 deficiency, *TANGO2* transport and Golgi organisation 2 deficiency. The definitions for fatty acid oxidation disorders abbreviations (in blue) are given in Table 1. Note that molecular testing should be preferred to muscle biopsy when available.

More typically, patients >4–6 years old present muscular symptoms. Exercise intolerance, with episodic rhabdomyolysis is common. Liver dysfunction may be present, and in some cases hypoglycaemic crises are triggered by prolonged fasting, cold exposure, strenuous physical exercise, or intoxication (e.g., accidental ingestion of alcohol). Finally, chorioretinopathy and peripheral neuropathy may be present in MTP/LCHAD, and can be the presenting and only symptom [4, 6]. Patients with at least one c.1528G > C allele in the *HADHA* gene, commonly develop severe retinopathy [5].

Long-chain FAOD (LC-FAOD) should be considered in any patient with cardiomyopathy, especially a new and acute onset. Here, biochemical testing of in vitro FAO flux or directly molecular genetic testing will allow a diagnosis to be made. Note that lactic acid is often mildly elevated in LC-FAOD during episodes of acute metabolic decompensation. For patients presenting with myopathic or neurological symptoms, plasma creatine kinase levels should be measured.

## Children > 11 years old and adults (Fig. 4)

Cases of late-onset FAOD often present with myopathic symptoms. The most frequent symptoms include exercise-triggered myalgia, rhabdomyolysis, cardiomyopathy, after prolonged fasting (>14 hours) or physiologic stress. Excessive consumption of alcohol during short period of time can also induce symptoms. Hypoglycaemia is less common than in younger patients. Retinopathy or sensory-motor axonal neuropathy are suggestive of MTP/LCHAD.

**Fig. 4:**
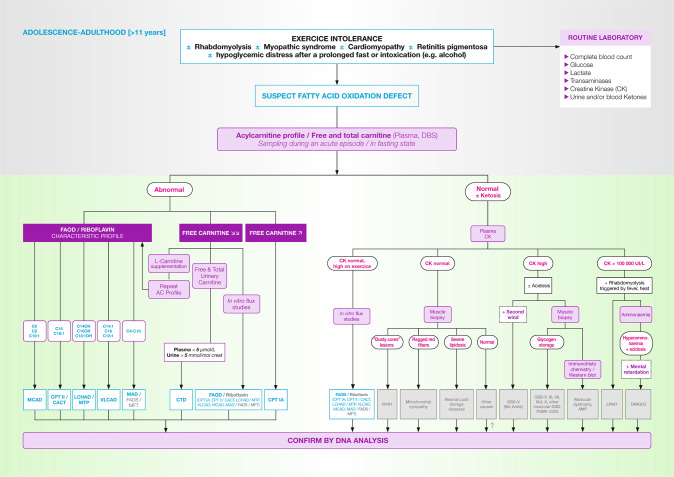
FAOD (fatty acid oxidation disorders) diagnosis in adolescents and adults. AC acylcarnitine, AMP adenosine monophosphate deaminase deficiency, CK creatinine kinase, DBS dried blood spot, FAD flavine adenine dinucleotide, FADS FAD synthase deficiency, GSD glycogen storage disorder, *LPIN1* phosphatidate phosphatase-1 deficiency, MFT mitochondrial FAD transporter deficiency, musc. GSD other muscular glycogen storage disorders, PGM1-CGD phosphoglucomutase 1 deficiency, *RYR1* ryanodine receptor 1 deficiency, *TANGO2* transport and Golgi organisation 2 deficiency. The definitions for fatty acid oxidation disorders abbreviations (in blue) are given in Table 1. Note that molecular testing should be preferred to muscle biopsy when available.

If the acylcarnitine profile is normal with elevated plasma creatine kinase levels in vitro flux studies or molecular genetic testing are helpful to reach a diagnosis. Muscle biopsies are non-diagnostic in FAOD.

## Post-diagnosis

Prompt diagnosis ensures immediate referral of patients to specialists and provides access to appropriate care and dietary management, thus reducing the risk of acute metabolic crises, while alleviating the chronic symptoms associated with FAOD. For example, intake of more frequent meals emphasising complex carbohydrates during illness, stress or increased activity, prevents acute decompensations in most FAOD [2]. Beyond dietary modifications, LC-FAOD (VLCAD [very long-chain acyl-CoA dehydrogenase], MTP/LCHAD, CACT [carnitine acylcarnitine translocase], CPT IA, CPT II) can be effectively and safely treated with an anaplerotic drug, triheptanoin. Triheptanoin has been shown to reduce episodes of myalgia, rhabdomyolysis, cardiomyopathy, and hypoglycaemia, along with emergency hospitalisations, leading to marked improvement in quality of life, but is not effective on retinopathy and neuropathy of LCHAD/MTP [6]. L-carnitine supplementation in CTD provides complete relief from symptoms. It may also be necessary in patients with severe secondary carnitine deficiency. SCHAD deficiency responds well to diazoxide [2].

Finally, genetic counselling can be proposed to families of an affected patient. Prenatal diagnosis is possible for all FAOD in chorionic villi or amniocytes, using molecular genetic testing as the preferred technique [2].

## Conclusion

The implementation of NBS for FAOD in more and more countries will most probably improve their diagnosis, but many patients still do not benefit from NBS. Moreover, it has been documented that NBS programs can miss diagnoses and that some patients can be symptomatic before the results of NBS are available [1, 4].

Considering this challenge, we hope that the presented tools will empower general practitioners working in a variety of settings to accelerate differential diagnosis for this group of metabolic disorders. More rapid FAOD diagnoses will improve the health and quality of life of patients, all of whom stand to benefit from the care available at specialised centres where their treatment may be adapted over the course of their lives. Even late onset, cryptic presentations of FAOD leave patients susceptible to potentially fatal metabolic crises. Through the reduction of hospitalisation rates due to acute metabolic decompensations, prompt and accurate referrals will also reduce burdens on local hospitals and associated costs to patients who do not receive appropriate treatment.
